# Sports injuries patterns in children and adolescents according to their level of sports participation, age and maturation

**DOI:** 10.1080/07853890.2021.1896775

**Published:** 2021-09-28

**Authors:** Lara Costa e Silva, Júlia Teles, Isabel Fragoso

**Affiliations:** a Laboratory of Physiology and Biochemistry of Exercise, FMH, Lisbon, Portugal; b CIPER, FMH, Lisbon, Portugal; c ESSATLA, Lisbon, Portugal; d Mathematics Unit, FMH, Lisbon, Portugal

## Abstract

**Introduction:**

Engaging in sports activities at a young age has numerous health benefits but also involves risk of injury. Growth can make young athletes more vulnerable to sports injuries [1]. Few studies have produced information about injury profile and its predictors at these ages. The aim of this study is to determine the influence of age, maturation and physical activity (PA) level on injury type and body area injury location on a Portuguese sample.

**Methods:**

A descriptive epidemiological study was conducted. Injury profile and PA level information’s were obtained by LESADO and RAPIL II questionnaires. These data allowed to create four groups of PA levels. The no sports participation group, with no time spent in PA per week (except mandatory physical education classes), the recreative sports group with at least 90 min of PA per week being at least 60% of this volume of recreational activity; the school sports group with at least 90 min of PA per week being at least 60% of this volume of school sports activity and the federated sports group with at least 120 min of federated activity. Maturity measures were evaluated through maturity offset and Tanner-Whitehouse III method. Univariate analysis was used to identify the set of candidate predictors for multinomial logistic regression analysis that was used to determine significant predictors of injury type and body area injury location. Ethics Committee of the Faculty of Human Kinetics approved the research protocol. Before inclusion in the study all subjects’ parents gave their written informed consent.

**Results:**

A total of 651 adolescents participated in this study, aged between 10 and 18 years (Mean = 13.7 years; Standard Deviation =1.8 years), being 343 boys (52.7%) and 308 girls (47.3%). Regarding injury type predictors recreative boys had more chances of having a sprain or a fracture than a strain when compared to federated boys (*χ*^2^(4)=15.165, *p*=.004). Also, recreative and scholar girls had more chances of having a sprain than a strain when compared to federated girls (*χ*^2^(6)=16.474, *p*=.011). As maturity offset decreased, the chances of girls having a strain or a fracture when compared to sprains were higher (*χ*^2^(2)=15.115, *p*=.001). For body area location boys with 10–11 years were more likely to have upper limbs injuries than boys of other ages (*χ*^2^(6)=13.587, *p*=.033). This was also confirmed by maturity offset (*χ*^2^(2)=6.014, *p*=.049). Spine and trunk injuries were more likely to occur in federate and no sports participation girls (*χ*^2^(6)=14.587, *p*=.022).

**Discussion and conclusions:**

Each sport group presented a specific injury profile and Peak Height Velocity was a significant predictor of injury patterns in adolescents of both sexes. The combination of growth, sport training and competition, create situations conducive to the development of specific injuries [2]. At these ages chronological age may be an incomplete indicator for injury risk, as some authors are starting to recognise [2,3]. It seems warranted that the influence of maturity status and PA level on sports injuries should be studied in future studies.
